# Reply: A paradox? Which paradox?

**DOI:** 10.1093/hropen/hoae007

**Published:** 2024-02-15

**Authors:** Edgardo Somigliana, Alessandra Chinè, Marco Reschini, Gianfranco Fornelli, Ludovica Basili, Andrea Busnelli, Paola Viganò, Ludovico Muzii

**Affiliations:** Infertility Unit, Fondazione IRCCS Ca' Granda Ospedale Maggiore Policlinico, Milan, Italy; Department of Clinical Sciences and Community Health, Università degli Studi di Milano, Milan, Italy; Department of Gynecology, Obstetrics and Urology, “Sapienza” University of Rome, Rome, Italy; Infertility Unit, Fondazione IRCCS Ca' Granda Ospedale Maggiore Policlinico, Milan, Italy; Department of Clinical Sciences and Community Health, Università degli Studi di Milano, Milan, Italy; Department of Clinical Sciences and Community Health, Università degli Studi di Milano, Milan, Italy; Department of Biomedical Sciences, Humanitas University, Pieve Emanuele, Milan, Italy; Division of Gynecology and Reproductive Medicine, Department of Gynecology, IRCCS Humanitas Research Hospital, Fertility Center, Rozzano, Milan, Italy; Infertility Unit, Fondazione IRCCS Ca' Granda Ospedale Maggiore Policlinico, Milan, Italy; Department of Gynecology, Obstetrics and Urology, “Sapienza” University of Rome, Rome, Italy

Sir,

We thank Drs Yang and Sun for their interest in our manuscript (Chinè *et al.*, 2023; Yang and Sun, 2024). In general, we fully share their comments and the emphasized limitations. Our study could not disentangle whether the effects of low ovarian reserve on the risk of miscarriage could be unremarkable also in older women and in those whose ovarian reserve has been injured by surgery. Other specific studies are required to clarify these two points.

Nevertheless, we would like to emphasize some aspects. The concept of remnant ovarian reserve must be distinct from the concept of oocyte quality. Since a pregnancy develops from one single oocyte, why should we expect that the process of folliculogenesis leading to the release of this oocyte could be of higher quality if it is accompanied by the growth of several rather than few other follicles? More in general, in our opinion, failing to observe a higher rate of miscarriage in women with lower biomarkers of ovarian reserve is not a paradox. It is logic. Why should we expect more miscarriages in women with lower biomarkers of ovarian reserve? We can expect a lower chance of success with IVF, but not a higher miscarriage rate. Older women tend to have a lower ovarian reserve and a higher risk of miscarriage, but the two may not be causally related. Ovarian reserve and oocyte quality decrease with age in parallel, but there are no logical reasons to postulate that they are intrinsically linked.

Unfortunately, designing studies that could provide a definitive answer to this question in older women is complex. Extremely large sample sizes are needed—maybe registers. For instance, we cannot repeat our study just recruiting women older than 35 years of age. This design cannot provide the answer. The detrimental effect of age on the risk of aneuploidies and miscarriage is mainly unremarkable up to the age of 35, then grows exponentially (Franasiak *et al.*, 2014; Magnus *et al.*, 2019). Age is so relevant that one should design a study like our one, but for every 1-year interval. Only registers could provide a sufficient sample size. Multivariate-adjusted analyses are an alternative, but they inevitably depend on arbitrary statistical models (thus possibly biasing the results). The meta-analysis from Busnelli *et al.* (2021) (that presented separately data from women younger and older than 35 years of age) could therefore not be informative. The analysis was too raw to protect findings from confounders.

Regarding the possibility that surgical damage could cause a different damage, i.e. causing a reduction in ovarian reserve that could be associated to a higher risk of miscarriage, we also deem this view implausible. A rationale to postulate that a detrimental effect on miscarriage rate could emerge only from oocytes obtained from operated gonads is lacking. Moreover, the subgroup analysis made in our study on operated women failed to support this possibility (even if the statistical power of this analysis was modest). Most importantly, there is evidence from several other studies suggesting that this may not be the case. The incidence of good-quality embryos from oocytes obtained from previously operated ovaries did not differ compared to intact gonads and the rate of aneuploidy is also similar (Somigliana *et al.*, 2023).

## Conflict of interest

The authors have no conflicts of interest to declare.
